# Seasonal Flight, Optimal Timing and Efficacy of Selected Insecticides for Cabbage Maggot (*Delia radicum* L., Diptera: Anthomyiidae) Control

**DOI:** 10.3390/insects3041001

**Published:** 2012-10-22

**Authors:** Renata Bažok, Mirna Ceranić-Sertić, Jasminka Igrc Barčić, Josip Borošić, Antonela Kozina, Tomislav Kos, Darija Lemić, Maja Čačija

**Affiliations:** 1 Department of Agricultural Zoology, Faculty of Agriculture, University of Zagreb, 10000 Zagreb, Svetošimunska 25, Croatia; E-Mails: akozina@agr.hr (A.K.); tkos@agr.hr (T.K.); dlemic@agr.hr (D.L.); mcacija@agr.hr (M.C.); 2 Agricultural Advisory Service, 47300 Ogulin, Bernardina Frankopana 13, Croatia; E-Mail: Mirna.Ceranic-Sertic@savjetodavna.hr; 3 Chromos-Agro d.d. 10000 Zagreb, Žitnjak bb, Croatia; E-Mail: Jasminka.Igrc-Barcic@chromos-agro.hr; 4 Department of Vegetable Crops, Faculty of Agriculture, University of Zagreb, 10000 Zagreb, Svetošimunska 25, Croatia; E-Mail: jborosic@agr.hr

**Keywords:** acetamiprid, cabbage maggot, dimethoate, dipping of the seedlings, degree-day accumulation, imidacloprid, Julian days, seasonal flight dynamics, thiamethoxam, yellow sticky traps

## Abstract

In order to describe seasonal flight activity of the cabbage maggot *Delia radicum* (L.) adults in relation to Julian days (JD), degree-day accumulations (DDA) and precipitation, flight dynamics were followed weekly with the use of yellow sticky traps (YST). Climatic data were collected and DDA were calculated using the lower developmental threshold of 4.3 °C. The efficacy of four insecticides applied either as standard foliar treatment or through dipping the seedlings before transplanting was determined. Seasonal flight activity during the cultivation season of a mid-early variety of white cabbage was correlated with DDA and JD and was characterized by having two peaks. The first peak occurred between 119 ± 7.5 JD and 125.5 ± 8 JD when DDA was 471.35 ± 74.97 °C. The second occurred between 172.8 ± 6.1 JD and 179.3 ± 6.7 JD when DDA was 1,217.28 ± 96.12 °C. The DDA, cumulative capture of flies and JD are suitable for predicting the timing of insecticide application. Spraying with insecticides should be applied when the cumulative capture of flies reaches 100 flies/YST and when DDA reaches 400 °C. If only one parameter reaches the threshold, additional visual surveys should be employed to establish the level of infestation. Insecticides were able to ensure only partial control. In the future, alternative control tactics which employ seed treatments and nonpesticide measures should be investigated in Croatia.

## 1. Introduction

Cabbage (*Brassica oleracea* L. convar. *Capitata* L.) production in Croatia is conducted on approximately 11,000 ha. Among all vegetables, cabbage is one of the most important. There are three main production regions, including north Croatia (in the vicinity of the cities of Varaždin and Čakovec), the Mediterranean region (along the Adriatic coast) and the mountain region (in the vicinity of the city of Ogulin). Among the pests in the region of Ogulin, the cabbage maggot, *Delia radicum *(L.), is the most important [1]. Although related species (*Delia floralis* Fallen and *Delia platura* Meigen) are listed as the members of the entomofauna of Croatia [2], there was no evidence that any other species except *Delia radicum* is damaging the cabbage of this region [1,2,3,4,5,6]. The agricultural area suitable for cabbage production in the region of Ogulin is limited. Crop rotation should reduce cabbage maggot infestations, but, the reality of cabbage production in this area is that cabbage is often grown continuously, which results in an increase in the cabbage maggot population.

There is limited information on cabbage maggot biology, ecology and control in Croatia [1,2,3,4,5,6]. The pest could develop three to four annual generations [1], with the largest populations arising from the overwintering of the first generation [4] compared to the other two generations. The overwintering generation impacts the production of early cabbage and has different emergence patterns in different regions of Croatia [1,2,3,4,6].

In Croatia, control of the cabbage maggot relies mostly on insecticide applications. This strategy is facing many problems related to a limited number of registered insecticides and a global decline in available active ingredients which may result in the banning of all organophosphorous (OP) insecticides in the next few years In Croatia, only three insecticides, chlorpyrifos, dimethoate and imidacloprid, are allowed to be used for cabbage maggot control [7]. A granular formulation of chlorpyrifos is applied as a band treatment before transplanting. Dimethoate and imidacloprid are systemic and are used either as a preventive measure by dipping of the seedlings and with irrigation after transplanting, or as a plant spray after the population is established. Vegetable growers in Croatia are under increasing pressure from the government to implement the principles of integrated pest management (IPM) into their production practices and to reduce pesticide use.

Preventive pesticide application is not in accordance with the principles of integrated pest management (IPM). However, dipping seedlings in insecticide solution during preplanting may be acceptable because this method localizes the insecticide application, which reduces the negative impact of insecticides on beneficial organisms and the environment. The foliar application of insecticides would result in a reduction in the number of pesticide applications only if it is based on supervised control and pest forecasting methods [8]. The advantage of forecasting systems is that they not only indicate the most appropriate times to apply insecticide treatments, but they could also be used to indicate when insecticide should not be applied.

The cabbage maggot overwinters as a pupa in the soil. The timing of spring emergence of adults and ovipostion activity depends on spring emergence patterns of cabbage maggot populations as a function of degree-day accumulation and can vary widely [9]. Forecasting systems should be based on an understanding of flight behavior of cabbage maggot adults and the relationships between adult flight activity, oviposition, crop damage and environmental conditions. Degree-day models have been widely used to assist in the development of control strategies for the cabbage maggot in many EU countries [10,11], the USA [9,12,13] and in Canada [14]. Most authors agreed that understanding temporal patterns of abundance is an important prerequisite for formulating appropriate management strategies and that regional risk forecasts need to take into account the emergence patterns of local populations. However, little phenological information [1,2,3,4,5,6] has been developed for this species in Croatia. 

The objective of this research was to describe and characterize seasonal flight activity of cabbage maggot adults in relation to Julian days, degree-day accumulations and precipitation as a key to improve monitoring and forecasting of the pest in early planted white cabbage in the region of Ogulin. In addition, we evaluated the efficacy of insecticides that were reported to provide good control of the cabbage maggot, but are not registered for the use in Croatia, compared to insecticides that are most frequently used to control cabbage maggot. Insecticides were applied either as standard foliar treatment or as a seedling dip before transplanting.

## 2. Materials and Methods

### 2.1. Experimental Sites

Investigations were conducted in 2007, 2008, 2009 and 2011, at trial sites located in the mountain part of Croatia (elevation 350 m), in the vicinity of Ogulin (latitude 45°15' N, longitude 15°13' E). The two basic studies were carried out. The experiments investigating seasonal flight activity and infestation were carried out on two fields (Field 1 and Field 2) each year except in 2011 when only one field (Field 1) was involved. The experiments investigating control were carried out on one field each year. These fields were in different parts of Field 1 and were planted separately. Seedlings were produced in a greenhouse in 0.33 × 0.5 m polystyrene containers each containing 208 seeds. Each seed was sown in 30 mL of potting medium (sterilized substrate “Klasmann” produced by Deilman). Seedlings were regularly fertilized by the application of the Kristalon Start and Albatros Sprint produced by Yara, Netherlands. Kristalon Starts contains 19% nitrogen, 6% phosphorous, 20% potassium and 3% magnesium, plus five microelements (boron, molybdenum, iron, copper, manganese and zinc). Albatros Sprint contains 10% nitrogen, 52% phosphorous and 10% potassium. Each fertilizer was applied twice by irrigation with the water solution that contains 0.15% fertilizer. Seedlings were transplanted into the fields six to seven weeks after sowing, when they had developed three to five leaves. The row spacing was 65 cm and the in-row distance between plants was 40 cm, such that the final plant density was 38,500 plants/ha. In 2011, the in-row distance between the plants was 33 cm *i.e.*, the final plant density was 46,620 plant/ha. Fields were maintained as free of weeds as possible by the use of herbicides. Herbicides (pendimethalin and metazachlor) were used as pretransplanting treatment and, if needed, either cycloxydim or fluazifop-P-butyl was used as posttreatment to control grasses.

### 2.2. Cabbage Maggot Flight Dynamics and Infestation

#### 2.2.1. Experimental Conditions

For experiments investigating flight dynamics and larval infestation, two fields each year were monitored between 2007–2009. In 2011, one field was monitored. The field size ranged between 0.26 and 0.43 ha, and the distance between the fields in 2007–2009 was less than 10 km. All fields were planted with the mid-early variety Krautman F1. Seedlings were transplanted using a transplanter between April 8 and April 24 (Table 1), and harvested between July 14 and August 14, depending on the year and the field. On experimental fields, no insecticides were applied and copper fungicides were applied to control downy mildew (*Hyaloperonospora parasitica*) only as needed. 

**Table 1:** Planting and harvesting dates and cultivation on fields involved in investigation.

Year	Field	Planting date	Harvesting date	Cultivation period (days)
2007	1	April 11	July 14	94
	2	April 19	July 14	86
2008	1	April 10	July 25	106
	2	April 18	August 1	105
2009	1	April 8	July 31	104
	2	April 24	August 14	112
2011	1	April 8	July 14	97

Climatic data (mean average air daily temperatures and daily precipitations) were collected by using the CDA ON LINE device (Agra d.o.o. Čakovec) which was set up in one of the trial sites. The device was calibrated to measure the air temperature three times per day and to recalculate the average daily temperature by dividing the sum of minimum and maximum by 2. From the collected climatic data starting 1 January, we calculated mean degree-day accumulation (DDA) by using the lower thermal threshold of 4.3 °C and upper thermal threshold of 30 °C as it was proposed by Derves *et al.* [9]. Cumulative precipitation also was calculated starting with 1 January.

#### 2.2.2. Flight Dynamics

Cabbage maggot flight dynamics were assessed using yellow sticky traps (Bio Plantella). The traps were a bright yellow, measured 210 by 297 mm and were covered on both sides with adhesive. The traps were held 1 m above ground level by attaching them with staples to wooden stakes. For consistency, each trap was placed at least 1.5 m from the edge of the plot. The minimal distance between the traps was 30 m.

Three yellow sticky traps were set up in each field on the same day seedlings were transplanted. The yellow sticky traps were changed weekly and removed from the field when the cabbage was harvested. The monitoring period was the same as the cultivation period and lasted between 86 and 112 days (Table 1). 

The yellow sticky traps were checked under the stereomicroscope and the captured cabbage maggot flies were counted for each inspection date. Data on the number of flies caught in a period of seven days were used to calculate the average daily capture of flies/trap. Also, fly captures on traps were accumulated until the end of the experiment for each year. 

#### 2.2.3. Infestation

In 2008, 2009 and 2011, every seven to ten days from planting until plants reached the developmental phase of copping, *i.e.*, the phase 6 in which plants developed 16–20 leaves and started to form the head [15], visual surveys were made on 100 plants in each of four rows. Plants were chosen randomly at the first survey and all following surveys were conducted on the same plants. Visual survey as a nondestructive method was proposed by Čamprag [16] to establish infestation of field crops and vegetable plants by pests. The same method is proposed in technical guidelines for integrated production of cabbage published by Ministry of Agriculture [17]. At each inspection date egg and larval density was established.

To establish egg density, on each sampling date 100 plants per row (*i.e.*, 400/field) were carefully examined for eggs visible around the plant stem base or on lower petioles [18,19]. The examination was carried out by moving the plant gently from side to side to examine soil surface and the gap between the plant stem and the soil. This was carried out without disturbing the soil. The number of visible eggs found around each plant was counted without removing them and the average number of eggs per plant calculated as proposed by Sekulić [20].

Plants affected by cabbage maggot appear stunted, off-color and took on a bluish cast. Severely damaged plants may wilt; plant wilting is particularly visible during the heat of the day. Therefore, in visual surveys, plants were inspected carefully and the percentage showing symptoms of larval infestation (wilting and change of color) recorded.

Starting on the survey date at which first symptoms of larval infestation were recorded, and at each subsequent survey, an additional 20 plants were chosen randomly (five per row) and dissected and the number of larvae and pupae per plant recorded.

#### 2.2.4. Statistical Analysis

The average daily captures of flies on yellow sticky traps were calculated for each period to determine flight dynamics. The average daily capture was calculated for each year and analyzed by ANOVA using ARM 7^®^ software (Gylling Data Management, Revision 7.2.2, 12 September 2005) with mean separation estimated using the Tukey HSD ranking test (*p* = 0.05) to establish the differences in populations among the years. Cumulative captures of flies on yellow sticky traps were correlated to DDA for each inspection date. Correlations were calculated using ARM 7^®^ software (Gylling Data Management, Revision 7.2.2, 12 September 2005). Regression equations were calculated using Microsoft Excel. To establish the differences among years, coefficients of determination and regression coefficients were analyzed by ANOVA using ARM 7^®^ software (Gylling Data Management, Revision 7.2.2, 12 September 2005) with mean separation estimated using the Tukey HSD ranking test (*p* = 0.05).

The number of eggs per plant and average percentage of plants showing the symptoms of larval infestation were analyzed regarding the Julian days, cumulative capture of flies and degree-day accumulations (DDA). Average numbers of larvae per plant were correlated to Julian days, to cumulative capture of flies on yellow sticky traps and to DDA for each inspection date in each year. 

### 2.3. Cabbage Maggot Control

#### 2.3.1. Experimental Conditions

Experiments investigating the efficacy of the insecticides were carried out in Field 1 in an area adjacent to, but separate from, the areas where investigations of seasonal flight dynamic and infestation were conducted. The seedlings were produced as described in 2.1. In 2007, the late cabbage variety Ogulinec was planted and in the other three years the mid-early variety Krautman F1 was planted. Fields were planted by hand. 

#### 2.3.2. Insecticides

The following insecticides were used: dimethoate (Chromgor 40—Chromos-agro d.o.o.) imidacloprid (Boxer 200SL—Chromos-Agro d.o.o.), thiamethoxam (Actara 25WG—Syngenta) and acetamiprid (Mospilan 20SP—Nippon).

All insecticides tested have a systemic mode of action [7]. Dimethoate is an organophosphate whereas imidacloprid, thiamethoxam and acetamiprid belong to the class of neonicotinoids. The neonicotinoids were developed in large part because they show reduced toxicity compared to organophosphate and carbamate insecticides. Most neonicotinoids show much lower toxicity in mammals than insects, but some breakdown products are toxic. In Croatia, dimethoate and imidacloprid are permitted for cabbage maggot control either as a preventive measure through dipping the seedlings and with irrigation after transplanting, or as a foliar spray after the cabbage maggot population is established. 

Insecticides were applied either by dipping the seedlings in containers of insecticide solution for 15 minutes one day before transplanting or by spraying according to the date established based on the observations of fly and egg numbers. Two days before dipping, plants were not irrigated and the blind probe with water was carried out in order to estimate the amount of insecticide solution that could be taken from each container. The amount of insecticide was calculated in order to achieve the optimal dose per ha. Treated and untreated seedlings were transplanted into the field the next day. Each treatment was applied on 4 plots, 25 m^2^ each. In each trial, an untreated control was involved. The treatments were arranged as randomized blocks. The foliar insecticide treatments were applied by means of a knapsack sprayer with 500–800 L of water/ha. The dates of insecticide application (Table 2) were determined by the numbers of flies on the YST, DDAs and visual inspection of the plants established on an adjacent field for the investigation of cabbage maggot flight dynamics and infestation. As proposed by Bažok and Ceranić-Sertić [3], insecticides were applied when the cumulative capture of flies was ≈100 flies/YST and when DDA reached 400 °C. If only one of the preconditions was fulfilled, a decision was made based on egg infestation. 

**Table 2:** Insecticides tested, rates and methods of application, and dates of application in each year.

Insecticide	Dose g a.i./ha	Application method *	Date of application			
			2007	2008	2009	2011
Dimethoate	400	DS			6 April	7 April
	400	SP	5 June	7 May	9 May	26 April
Imidacloprid	167	DS	6 May			
	200	DS			6 April	7 April
	100	SP	5 June	7 May	9 May	26 April
	200	SP				26 April
Thiamethoxam	200	DS	6 May	8 April	6 April	
	100	SP	5 June	7 May	9 May	
Acetamiprid	46	DS	6 May			
	50	DS			6 April	
	50	SP		7 May	9 May	

* DS = dipping of the seedlings into insecticide solution before transplanting; SP = spraying.

#### 2.3.3. Assessments

The all experimental plots were inspected regularly to establish the date when the first plants showing symptoms of larval infestation (wilting) appeared. After that date, every seven to ten days all plants on each experimental plot were evaluated, as described in 2.2.3. The presence of cabbage maggot was assessed six weeks (2008), and seven weeks (2009 and 2011) after planting by removing ten randomly chosen plants per plot. The larvae and/or pupae were counted on each plant, in the roots and in the part of the stem that was below the ground as proposed by Ester *et al*. [21].

#### 2.3.4. Statistical Analysis

All results were analyzed using ANOVA. Tukey’s HSD tests were used to determine the differences between the mean values (*p* = 0.05) (ARM 7 GDM software, Revision 7.2.2. 2005).

## 3. Results and Discussion

### 3.1. Experimental Conditions

Total amounts of precipitation between 1 January and 14 July were 688.5 mm, 650.9 mm, 724.3 mm and 499.5 mm, for 2007, 2008, 2009 and 2011 respectively. This is less than the 30-year average for the same period, *i.e.*, 858.6 mm [22]. The amounts of precipitation which were received before the transplanting of cabbage seedlings in 2007, 2008, 2009 and 2011 were 371.7, 374.9, 484.0 and 195.2, respectively. Except for 2011, this amount is close to the 30-year average for that period (≈400 mm). The total amounts of precipitation during the growing season varied slightly between 316.6, 276.0, 240.3 and 304.3 mm in 2007, 2008, 2009 and 2011, respectively. These amounts are much lower than the long-term average for the same period (≈450 mm). The distribution of precipitation during the growing season (April to July) differed among years, and was below average in April and May in 2007 and in May 2009 (Table 3).

**Table 3:** Mean monthly temperature and precipitation data during the growing season at Ogulin, Croatia, in 2007–2009 and 2011 (data obtained from the CDA device) compared with the long-term average [14].

Month	Temperature (°C)					Precipitation (mm)				
	2007	2008	2009	2011	1961–1990	2007	2008	2009	2011	1961–1990
January	6.3	3.7	−1.7	1.8	−0.5	150.9	73.9	150.9	70.7	105.8
February	6.2	5.3	2.2	0.7	1.4	132.2	38.6	119.9	38.0	109.8
March	7.8	6.0	6.1	5.9	5.1	126.7	202.3	138.6	64.2	122.3
April	12.9	10.6	13.1	12.2	9.6	12	102.5	193.3	83.7	137.6
May	15.6	15.4	17.2	15.1	14.2	43.8	74.1	23.4	111.3	124.7
June	19.7	19.2	18.1	19.1	17.4	103.9	115.9	48.1	126.8	129.3
July	20.9	20.1	21.2	20.6	19.2	145.9	79.1	62.6	138.4	129.3

However, precipitation patterns during the growing season in 2008 and 2011 were much closer to long-term mean values. Mean temperatures during these periods in all years of investigation were above the long-term averages, except the mean monthly temperature in January 2009. Depending on the month and year, temperatures ranged from 0.7 °C to 3.5 °C higher than the long-term average. Total degree-day accumulations at air base 4.3 °C for 2007, 2008, 2009 and 2011 were 1,598.8, 1,728.7, 1,969.0 and 1,498.0 °C, respectively. The differences between the years were partially caused by the fact that monitoring periods were not the same in all four years. The monitoring period finished on July 14 in 2007 and 2011, but in 2008 and 2009, it finished on 1 August and 14 August, respectively.

### 3.2. Cabbage Maggot Flight Dynamics

There was no literature to support that any other *Delia* species is damaging to cabbage in the region of Ogulin and the flies on the YST were identified to genus level only. Workers from Croatia (1–6) always mentioned *Delia radicum* as the main pest of cabbage and this species is always mentioned as the most serious pest of the brassica crops in Western Europe [8,19,21]. Additionally, Sandrine *et al.* 2006 [23] indicated that the distribution of the most similar species, *Delia floralis* is more restricted to Northern regions with lower temperatures and does not exist at latitudes below 50° N. Therefore, all *Delia* flies on the traps were considered as *D. radicum*.

Populations of cabbage maggot flies were much lower in 2007 than in the other three years. The average captures of the files per trap/day in 2007, 2008, 2009 and 2011 were 2.45, 10.64, 7.84 and 13.55, respectively. The differences were significant (LSD *p* = 0.05 = 3.28). Despite the differences in average captures, populations could still be considered high in all years. Other authors reported maximal daily captures of between 1 and 14 flies [12] and a maximal weekly capture of 8–15 flies per trap [12]. However, they used trap size that was double as small.

**Figure 1:**
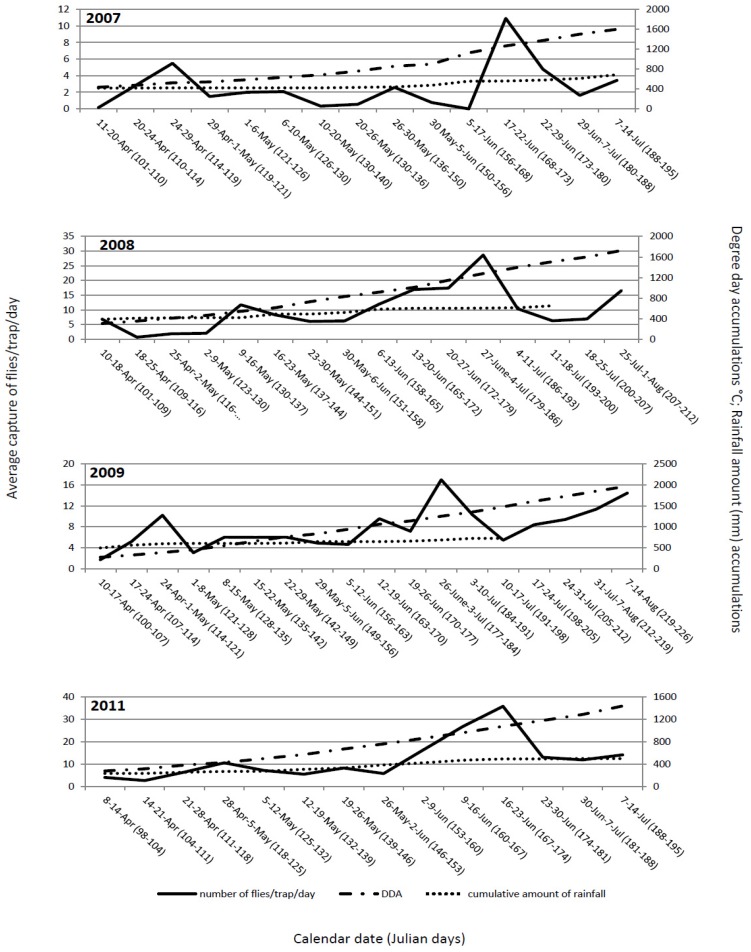
*Delia radicum* (L.) adult captures per trap per day on yellow sticky traps in early cabbage fields in Ogulin, Croatia compared with DDA (base 4.3 °C base threshold), and the cumulative amount of precipitation over four field seasons (2007 to 2009, 2011).

The flight dynamics in all four years mainly showed similar patterns. The first flies were observed immediately after placing out yellow sticky traps, suggesting that at the time of transplanting, the flies had already emerged from their overwintering sites and, immediately after cabbage was transplanted, migrated to the new fields. Generally, the flight dynamics had two peaks: one at the end of April and beginning of May and, the second between mid and the end of June (Figure 1). These peaks probably correspond to two generations. The peak of the flight of overwintering cabbage maggot flies could be predicted with 15 to 16 days of delay or with ≈150 °C difference in DDA. It starts at 119 ± 7.5 Julian days and ends at 125.5 ± 8 Julian days. At that time, degree-day accumulation reaches 471.35 ± 74.97 °C. Some authors [14,24] stated that even though DDA could predict fly emergence and activity with reasonable accuracy, the calendar date was comparable or better than DDA as a predictive tool. Our results do not support that the calendar date is better than DDA as a predictive tool. Transplanting early cabbage in the open fields in the region of Ogulin is regularly conducted between April 10 and 20. Obviously, the emergence of the flies occurs earlier and, at the time of transplanting, the flies are already emerged and ready to migrate to the newly planted cabbage fields. The importance of DDA is related to the conditions for fly emergence and the numbers of flies present at planting. The peak of the flight of the second generation could be predicted with a delay of 12 to 13 days or with ≈190 °C difference in DDA. It started between at 172.8 ± 6.1 Julian days and ended at 179.3 ± 6.7 Julian days. At that time, degree-day accumulations were 1,217.28 ± 96.12 °C.

In Oregon, which has the similar latitude as Ogulin but a lower elevation, cabbage maggot shows a bimodal spring emergence pattern. Bimodal spring emergence patterns may be driven by genetic variability, *i.e*., population composition [25]. Waagelbach *et al*. [26] showed that cabbage maggot populations consist of different ratios of early and later emerging individuals. If one biotype prevails, the population will not show a bimodal emergence pattern. For northern European populations, Finch and Collier [27] found both biotypes, but large variation in the percentage of each biotype in local populations. We do not have data on the makeup of the fly population in the region of Ogulin, but if we compare our data on degree-day accumulations needed for the spring flight, we can conclude that in this region, one biotype prevails and it is most likely the early emerging biotype. There is evidence that in the region of Međimurje (northeast of Ogulin), the cabbage maggot adult shows different emergence and flight patterns [6] compared to Ogulin. Therefore, if we wish to better understand emergence patterns in different regions of Croatia, additional research is needed, particularly to determine the developmental thresholds and to establish the percentage of early/late flies. As was suggested by Turnock and Boivin [25], the results may indicate if the percentage early/late remains quite constant or changes over time in each particular region.

**Figure 2:**
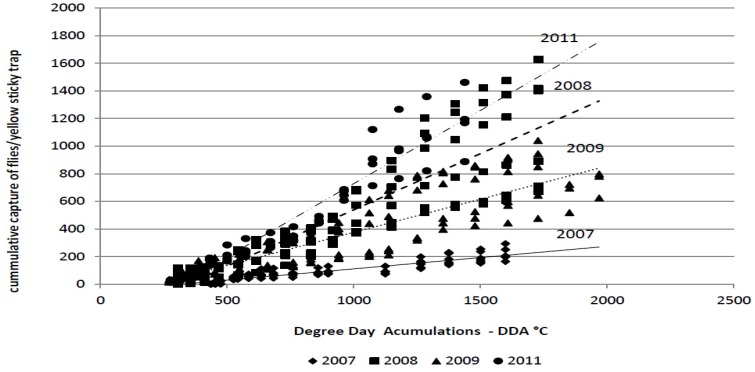
Linear regression analysis of the degree-day accumulations (base threshold 4.3 °C) *vs.* the cumulative capture of the cabbage maggot adults (*Delia radicum* (L.))on yellow sticky traps over four field seasons (2007 to 2009, 2011).

The average cumulative captures of the flies during the monitoring period in 2007, 2008, 2009 and 2011 were as follows: 220.32; 1,121; 840.33; and, 1,178.5 flies/trap, respectively. The cumulative captures depend on degree-day accumulations, *i.e.*, temperature (Figure 2). The spring flight of the flies in cabbage fields was observed soon after transplanting in all years. At that time, degree-day accumulation had reached 270–435 °C.

The amount of variability was measured using the coefficient of determination (R^2^) (Table 4). The coefficient of determination indicates that the cumulative capture of flies on yellow sticky traps could be accurately predicted by the DDA in all years of the study (R^2^ = 0.933–0.977). The significant influence of the cycle, *i.e.*, weather conditions, on the regression line is clear. The regression coefficient and the coefficient of determination were significantly lower in 2007 compared with all other years. 

**Table 4:** Data obtained in the linear regression analysis (coefficients of determination (±SE), probability, *n*, and regression coefficients (±SE) of the degree-day accumulations (above 4.3 °C and below 30 °C) (x) *versus* the cumulative number of *Delia radicum* (L.) flies /yellow sticky trap (y) over four field seasons (2007 to 2009, 2011).

Year	Coefficient of determination R^2^ (±SEM)	n **	p	Regression coefficient b (±SEM)	Average daily capture of flies per trap/day
2007	0.933 ± 0.017 b *	96	0.0001	0.163 ± 0.036 c	2.45 c
2008	0.977 ± 0.0035 a	96	0.0001	0.814 ± 0.326 ab	10.64 ab
2009	0.971 ± 0.182 a	96	0.0001	0.541 ± 0.134 b	7.84 b
2011	0.965 ± 0.024 a	56	0.0001	1.059 ± 0.240 a	13.55 a
LSD_p = 0.05_	0.019			0.275	3.28

* Means followed by the same letter are not significantly different according to Tukey’s HSD test (*p* = 0.05). ** number of data sets involved in the analyze.

The linear regression analysis showed that there is significant impact of the year of the study, on the slope of the regression line, *i.e.*, on the regression coefficient (b). It was the lowest in 2007. April of 2007 was extremely dry and very warm. As the mean monthly temperatures in January, February and March in 2007 also were above average, it is possible that the flies emerged very early. It is known that cabbage maggot development may be stimulated by rainfall [28]. After emergence, flies feed on pollen and nectar produced by various plants [9], and start to mate and lay eggs approximately one week after emergence. Cabbage maggot adults deprived of host plants are less selective in choosing oviposition sites compared to females able to continuously lay eggs on host plants [29]. In 2007, cabbage was transplanted as usual in April. It is possible that the flies had emerged earlier than the transplanting date in 2007, were deprived of host plants and therefore were less selective in choosing oviposition sites than in typical years.

Regression coefficients were higher in the years when the total numbers of adults captured on yellow sticky traps were higher. It is expected because the higher number of adult means larger sample what result in highest accuracy and lower amount of variability.

### 3.3. Cabbage Maggot Infestation

**Figure 3:**
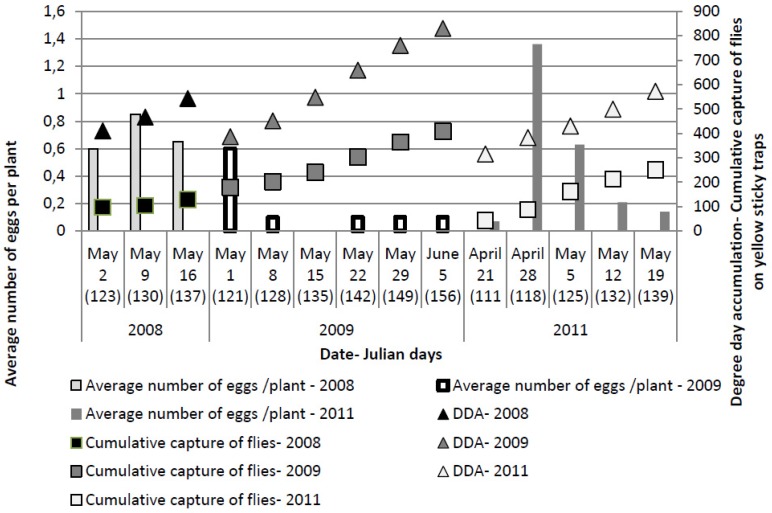
Numbers of *Delia radicum* (L.) eggs per plant on early cabbage, compared with cumulative numbers of *D. radicum* adults on yellow sticky traps and DDA over three field seasons (2008, 2009, 2011).

The first egg laying was observed two to three weeks after transplanting, with the first eggs found on May 2, May 1 and April 21 in 2008, 2009 and 2011, respectively. Although an average of up to four eggs were observed on individual plants, because some plants had no eggs, the average number of eggs per plant was between 0.6 and 0.85 in 2008 and up to 0.63 in 2009, respectively. The only exception was on April 28, 2011 when 1.36 eggs/plant were recorded (Figure 3). Egg numbers were low compared to those of Easter *et al*. [30] who reported 13 or more eggs per plant. Bligaard [31] noted that an artificial infestation with 100 eggs per plant resulted in a 5% reduction in plant growth. Bligaard [18] considered an average infestation of 21 eggs/infested plant, with 30% of plants infested, as an economic threshold (ET). If the same infestation is recalculated into the average number of eggs per plant, the economic threshold is 6.2. The highest average number of eggs/plant in our trial was 1.36, less than the ET discussed by Bligaard [18]. In the guidelines for cabbage production published by Ministry of Agriculture Republic of Croatia [17] an ET of one to two eggs or larva/plant is recommended, much lower than the ET suggested by Bligaard [18]. The difference is probably related to sampling method; Bligaard [18] presented results obtained by absolute sampling in which 99% of eggs could be obtained, whereas the guidelines for cabbage production in Croatia recommend a nondestructive method, *i.e.*, a visual survey of the plant base, which underestimates the number of eggs present. Although eggs are largely deposited in the upper soil layer by the stem base or on the lower petioles, under natural conditions, the female fly pushes its ovipositor into the soil, leaving only a few eggs exposed on the soil surface and others only partly visible or buried. Therefore this method is less precise than an absolute sampling method.

The highest number of eggs per plant (Figure 3) was recorded at 123 ± 6 Julian days over the three years, which corresponds with the end of April and beginning of May. At this time, the cumulative captures were 124.11 ± 48.6 flies per yellow sticky trap and DDAs had reached 412.31 ± 47.34 °C.

**Figure 4:**
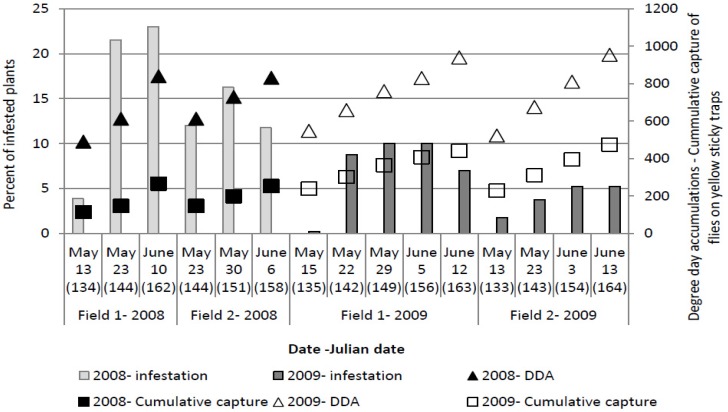
The average percent of plants showing symptoms of larval infestation by *Delia radicum* (L.) over two field seasons (2008, 2009).

Although in 2011, the number of eggs was similar to or higher than that observed in the other two years, no plants showing symptoms of larval infestation were recorded in the visual survey. A higher percentage of plants showing symptoms of larval infestation (wilting) was observed in 2008, compared to 2009 (Figure 4). The experimental field in 2011 was not rich with organic matter, so it is likely that the eggs were *D. radicum* rather than a related species like *Delia platura*, which lays eggs similar in appearance to those of *D. radicum*, and which feeds on organic matter in the soil. There are several possible causes for the absence of symptoms of wilting in 2011: egg and larval mortality, presence of natural enemies, or ability of the plants to tolerate a certain number of larvae. Bligaard [31] reported that the percentage of mortality of eggs and larvae is very high (between 47% and 61%). Eggs of *D. radicum *are resistant to low soil moisture and high temperature conditions, while larval survival tends to increase with an increase in soil temperature up to 33 °C and in the and moisture [32]. Climatic conditions during the period of egg laying and larval development (April and May) in 2011 were similar to those observed in the other three years, which supports the idea that the absence of wilting symptoms was due to other factors. In May of 2011 the amount of precipitation was the highest among all experimental years, and this may have influenced larval survival [32]. Several authors [8,33,34] have suggested that some of the 60–100 species of carabid and staphylinid beetles found commonly in cultivated soils are important predators or parasitoids of the cabbage maggot and that their activity is related to crop rotation and cultivation practices. In fact, over the three years of investigation the highest number of larvae per plant was found in 2011. It is possible that the low percent of the plants showing symptoms of larval infestation was due to the very good growing conditions in 2011, which enabled plants to develop very quickly and successfully even in the presence of larvae.

The maximum number of plants which showed symptoms of larval infestation was recorded on 153 ± 6 Julian days, which corresponds with the end of May and beginning of June. At the time when the highest number of plants showing symptoms of larval infestation was observed, cumulative captures were 299.07 ± 109.07 flies per yellow sticky trap and DDAs had reached 764.1 ± 85.15 °C.

**Figure 5:**
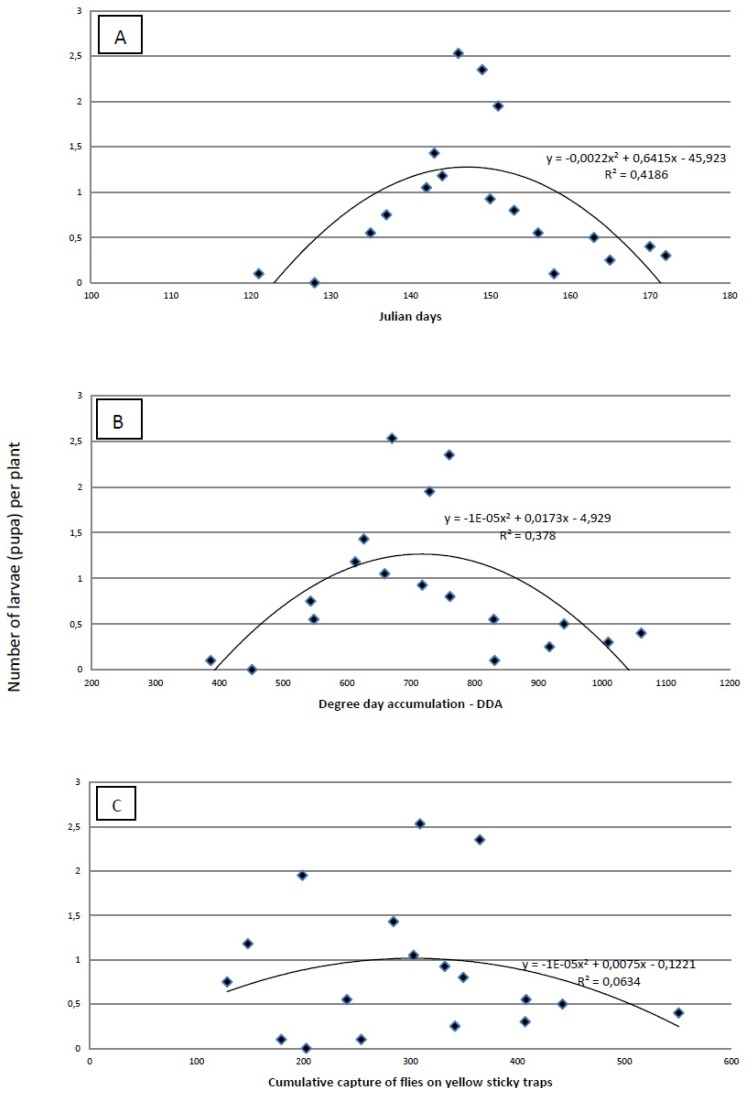
Regression analysis of Julian days (**A**), degree-day accumulation (base threshold 4.3 °C)—DDA (**B**) and cumulative capture of cabbage maggot flies on yellow sticky traps (b) *versus* the average number of cabbage maggot larvae)/plant over three field seasons.

The proof that a visual survey of the plants for eggs is less precise than absolute sampling is obvious from the results of plant dissections. Using dissection, we found the highest infestation was 1–2.5 larvae per plant (Figure 5), but the highest number of eggs found by visual survey was 1.36 eggs/plant. The maximum number of larvae per plant was recorded on 146 ± 3 Julian days, which corresponds to the end of May. At the time when the highest number of larvae per plant was established, cumulative captures were 267.99 ± 79.64 flies per yellow sticky trap and DDAs had reached 676.73 ± 57.80 °C. The most accurate tool for predicting the maximum number of larvae/plant is Julian days. The amount of variability was measured using the coefficient of determination (R^2^) (Figure 5). This indicates that the maximum number of larvae per plant was better predicted by Julian days (R^2^ = 0.4186) and by the DDA (R^2^ = 0.378) than by the cumulative capture of flies on yellow sticky traps (R^2^ = 0.0634).

The mean larval damage occurred between the period when the maximum number of eggs were observed and the period when maximum symptoms of larval infestation were visible. If cabbage maggot is controlled by foliar application of insecticides, the application should be conducted a few days after the maximal number of eggs is established and before the maximal number of larvae is established. The data obtained in our investigation could serve as the parameters to help to determine the optimal timing of foliar insecticide application.

### 3.4. Cabbage Maggot Control

The trial in 2007 was carried out with the late variety Ogulinec which was planted in beginning of May, approximately 40 days after the peak flight of the overwintering generation was recorded. Late planting resulted with a low attack of cabbage maggot (Table 5) because the most vulnerable stage of the plants corresponds with the period between two generations of cabbage maggot flies. Although the peak flight of the first generation was recorded between 17 and 22 June (Figure 1), we did not establish high larval infestation in the second part of the trial. Larval infestation was not high enough to cause significant damages on the plants. At the beginning of July, when larvae from this generation started to infest the plants, plants reached the phenological phase of copping [15], *i.e.*, they began to form a head and thus probably became more tolerant to larval infestation as it was stated by Bligaard [31]. Established infestation confirmed the statements of some authors [2,5] that the early cabbage varieties are more endangered from cabbage maggot infestation.

**Table 5:** Percentage (±SEM) of cabbage plants showing symptoms of *Delia radicum* (L.)larval infestation (wilting) in 2007 following treatment with various insecticides, or untreated.

Treatment	Dose g a.i./ha	Appl. method *	Date of evaluation			
			22 June	29 June	6 July	13 July
Untreated			5.0 ± 4.08	7.5 ± 2.89	2.5 ± 2.89 ab**	1.88 ± 3.75
Dimethoate	400	SP	6.25 ± 7.5	5.63 ± 4.73	5.0 ± 2.04 ab	0
Imidacloprid	167	DS	10.0 ± 8.16	10.0 ± 8.16	2.5 ± 2.89 ab	5.0 ± 4.08
	100	SP	1.88 ± 1.25	1.25 ± 1.44	3.75 ± 2.5 ab	0
Thiamethoxam	200	DS	5.0 ± 5.77	10.0 ± 8.16	2.5 ± 2.89 ab	5.0 ± 4.08
	100	SP	3.75 ± 5.95	5.0 ± 5.4	8.13 ± 5.15 a	0.63 ± 1.25
Acetamiprid	46	DS	0	2.5 ± 2.89	0 b	0
LSD _p = 0.05_			ns	ns	6.61	ns

* DS = dipping of the seedlings into insecticide solution before transplanting; SP = spraying; ** Means followed by the same letter are not significantly different according to Tukey’s HSD test (*p *= 0.05).

The highest percent of plants showing symptoms of wilting, among the all years was observed in 2008. Up to 20.75% of plants on untreated plots had been damaged by larvae (Table 6).

**Table 6:** Experimental results determined on the basis of visual survey of plants and observing percent of plants showing symptoms of *Delia radicum*(L.)larval infestation (±SEM) in 2008 following treatment with various insecticides, or untreated.

Treatment	Dose g a.i./ha	Appl. method *	Date of evaluation			
			12 May	23 May	7 June	17 June
Untreated			3.75 ± 2.63	20.75 ± 2.06 a**	19.5 ± 7.14	19.75 ± 10.14
Dimethoate	400	SP	2.25 ± 0.5	13.75 ± 2.06 b	18.5 ± 3.7	20.0 ± 4.24
Imidacloprid	100	SP	3.0 ± 1.41	13.5 ± 2.06 b	16.5 ± 6.56	15.25 ± 5.74
Thiamethoxam	200	DS	1.5 ± 0.58	13.5 ± 1.91 b	16.75 ± 11.53	16.25 ± 10.37
	100	SP	3.5 ± 1.91	13.0 ± 1.26 b	19.75 ± 4.35	19.5 ± 7.55
Acetamiprid	50	DS	1.5 ± 0.58	7.0 ± 4.11 c	10.75 ± 4.92	10.5 ± 4.36
LSD _p = 0.05_			ns	5.7	ns	ns

* DS = dipping of the seedlings into insecticide solution before transplanting; SP = spraying; ** Means followed by the same letter are not significantly different according to Tukey’s HSD test (*p *= 0.05).

The percent of plants showing symptoms of wilting in 2009 was much lower than in 2008. A maximum of 5.25% of plants on untreated plots and 9.00% on acetamiprid applied as a seedling dip had been damaged by larvae (Table 7).

**Table 7:** Experimental results determined on the basis of visual survey of plants and observing percent of plants showing symptoms of *Delia radicum* (L.) larval infestation (±SEM) in 2009 following treatment with various insecticides, or untreated.

Treatment	Dose g a.i./ha	Appl. method *	Date of evaluation			
			13 May	23 May	3 June	14 June
Untreated			1.75 ± 1.5	3.75 ± 2.22 ab**	5.25 ± 1.89	5.25 ± 1.26
Dimethoate	400	DS	0.75 ± 0.96	4.5 ± 3.7 ab	5.25 ± 4.19	3.75 ± 3.59
	400	SP	0.25 ± 0.5	2.5 ± 1.0 b	3.0 ± 1.41	3.0 ± 1.15
Imidacloprid	200	DS	0.25 ± 0.5	3.0 ± 3.56 ab	2.75 ± 3.59	2.25 ± 2.63
	100	SP	0.5 ± 1.0	2.25 ± 1.5 b	2.75 ± 3.1	2.0 ± 1.41
Thiamethoxam	200	DS	0.75 ± 1.5	2.5 ± 2.65 b	2.0 ± 3.57	1.5 ± 2.38
	100	SP	1.5 ± 1.29	4.0 ± 1.41 ab	6.75 ± 2.5	2.25 ± 2.22
Acetamiprid	50	DS	3.5 ± 2.65	9.0 ± 5.03 a	6.0 ± 4.9	5.25 ± 4.11
	50	SP	0.25 ± 0.5	1.75 ± 2.36 b	2.5 ± 2.08	2.0 ± 1.26
LSD _*p* = 0.05_			ns	6.05	ns	ns

* DS = dipping of the seedlings into insecticide solution before transplanting; SP = spraying; ** Means followed by the same letter are not significantly different according to Tukey’s HSD test (*p *= 0.05).

In visual surveys conducted on May 2, 17, 23, and 30, 2011, very few plants showed symptoms of wilting (up to 1.5%). Therefore the results were not processed by ANOVA.

The data on the number of larvae per plant (Table 8) show the opposite with regards to larval feeding, compared to the data from visual surveys. This indicates that observing plant damage without establishing the number of larvae per plant could result in an erroneous conclusion. Plants which had symptoms of wilting and had changed color were infested with different numbers of larvae. Other factors such as temperature, humidity and agronomic conditions may influence the final effect of the same larval infestation on plant development and survival. Researching insecticide efficacy and damage thresholds, other workers [30,32] used the establishment of the following three things as criteria: the number of larvae and pupae/plant; the number of dead plants; and, the dry matter content in the roots. However, only by determining the dry matter content could the difference between plants which were infested but which were able to compensate larval damage, be established. 

Among all years, the highest number of larvae per plant was observed in 2011. The fact that high adult populations also were observed in 2011, but with only a few plants with symptoms of wilting, supports our conclusion that in 2011, good cultivation conditions such as adequate precipitation and favorable temperatures enabled plants to tolerate the larval infestation. Ester *et al.* [30] also reported differences between years in the response of cauliflower plants to similar larval infestations. They suggested that when it is wet, plants develop new roots permitting rapid regrowth following insect feeding. In our investigation, the difference in amount of precipitation between 2008 and 2011 in May when main larval damage occurred (Table 3) was not very high. However, the amount of precipitation was somewhat higher in 2011 and this, together with better plant conditions and very good fertilization, contributed to better plant regrowth in 2011 compared to 2008. It should be noted that white cabbage plants have a stronger capacity for plant regrowth compared to cauliflower plants [30].

**Table 8:** Experimental results determined on the basis of dissection of plants and establishing average number of *Delia radicum* (L.) larvae/plant (±SEM) in three year trials with insecticides, or untreated.

Treatment	Dose g a.i./ha	Application method *	Average number of larvae/plant ±SEM		
			2008	2009	2011
Untreated			1.33 ± 0.83 ab**	1.8 ± 0.2 bc	2.2 ± 0.36 ab
Dimethoate	400	DS		0.5 ± 0.12 f	1.38 ± 0.62 bc
	400	SP	1.48 ± 0.56 ab	1.53 ± 0.29 cd	1.08 ± 0.25 cd
Imidacloprid	200	DS		2.9 ± 0.73 a	0.25 ± 0.13 d
	100	SP	1.23 ± 0.86 ab	0.75 ± 0.93 ef	0.58 ± 0.17cd
	200	SP			2.85 ± 0.79 a
Thiamethoxam	200	DS	0.38 ± 0.29 b	1.3 ± 0.08 cde	
	100	SP	0.43 ± 0.53 b	0.93 ± 0.05 def	
Acetamiprid	50	DS		2.29 ± 0.24 ab	
	50	SP	1.92 ± 0.9 a	0.88 ± 0.17 def	
LSD *_p _*_= 0.05_			1.43	0.73	0.95

* DS = dipping of the seedlings into insecticide solution before transplanting; SP = spraying; ** Means followed by the same letter are not significantly different according to Tukey’s HSD test (*p *= 0.05).

**Figure 6:**
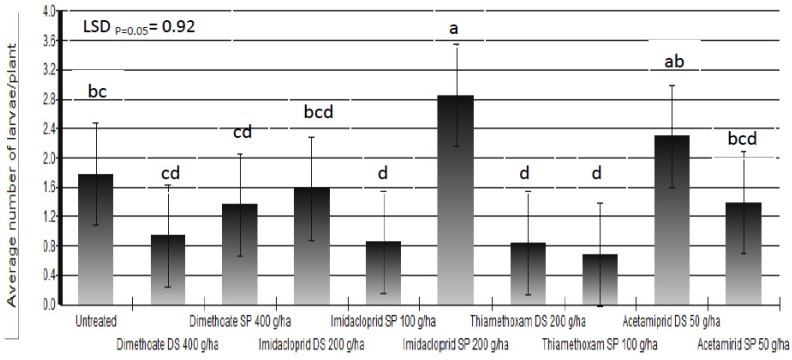
Average number of *Delia radicum* (L.) larvae/plant following different insecticide treatments (DS = dipping of the seedlings into insecticide solution before transplanting; SP = spraying)over three field seasons (means followed by the same letter are not significantly different according to Tukey's HSD test (LSD*_p _*_= 0.05_ = 0.92).

Based on the results of the three-year trials we could not state that any of the applied insecticides protected white cabbage plants completely against cabbage maggot larvae. The percent of plants which showed symptoms of larval infestation was reduced up to 67% in insecticide-treated plots compared to untreated control plots. Beside the larval infestation, the final damage was determined by additional factors which influenced plant regrowth. Somewhat better results were achieved in the reduction of average number of larvae/plant (Figure 6). There are three possible causes of poor insecticide efficacy: leaching or degradation of insecticides; incorrect timing of foliar application of insecticides; and, lack of efficacy of applied insecticides.

By applying insecticides as seedling dips, we apply them before larval infestation occurs. Insecticides enter the potting mix first and, if they are systemic (as it was the case in our trials), they are immediately taken from the plants. The amount of insecticides that is taken from the plants depends on various factors (plant size, plant water intake, *etc*.). However, a portion of the active ingredient could be retained in the potting mix and be taken from the plant later. Leaching of insecticides from the potting mix and degradation of insecticides in both, potting mix and plants, could occur in the period between transplanting and larval infestation. For good activity, the amount of insecticide in the plant tissues has to remain at the toxic level up to the time when larval infestation occurs. The time span between transplanting (*i.e.*, application) and the time when the maximum larval attack occurs influences the insecticide efficacy. The important factor is probably the amount of rainfall in this period. Rainfall could increase leaching of insecticides from the upper soil level or could influence the speed of breakdown of applied insecticides. In 2008, there was a time span of 40 to 50 days between insecticide application and peak larval infestation and the amount of precipitation in April and May was very high. This could have led to relatively poor results of insecticides applied as a dipping of the seedlings. Compared to 2008, the time span between transplanting and larval infestation was longer in 2009 but the amount of rainfall in this period was somewhat lower (especially in May). In 2011, a high amount of rainfall after transplanting was recorded, similar to 2008.

Dimethoate applied as a seedling dip showed better results in 2009 compared to 2011, probably because it is highly soluble in water, it adsorbs only very weakly to soil particles and thus it may be subject to considerable leaching [35]. It is known that dimethoate breaks down faster in moist than in dry soils and is rapidly broken down by most soil microorganisms. On the other hand, imidacloprid breaks down within one year [36]. It is immobile in the soil and, according to Lee *et al*. [37], after it is applied it stays in upper soil levels (root zone). When applied as a seedling dip, imidacloprid showed better efficacy in 2011 than in 2009.

Thiamethoxam has no bioaccumulation potential in the soil [38]. It degrades into other neonicotinoid insecticide, chlothianidin [39].

However, the main portion of insecticides at the time of transplanting was already taken from the plants and is thus not exposed to leaching. The residual activity of the various applied insecticides is similar [38,39,40,41]. 

At the time of foliar application, plants were small and insecticides were applied on both the soil surface and the plants. The insecticide that was applied to the soil surface could have been exposed to leaching or photodegradation. In 2008, in the second week following foliar application of insecticides, a large amount of rainfall was recorded (69.00 mm/m^2^). Similar climatic conditions were recorded in 2011; in the two weeks following foliar application in 2011, 35.0 mm rain/m^2^ was recorded. In 2009, no rain was recorded in the two-week period that followed foliar application. However, it could not be concluded that amount of rainfall after foliar application of any of applied insecticides influenced their activity. After application, dimethoate is rapidly absorbed and decomposed both on the surface and inside the foliage [35], and, on the soil surface, imidacloprid degrades very quickly [37]. Thiamethoxam is moderately mobile in soil and degrades at fast to moderate rates under field conditions [38]. The quick photodegradation of acetamiprid was noted by Gupta *et al*. [42].

The one reason for poor insecticide efficacy could be the timing of the application. The foliar application of insecticides corresponded with a maximum number of eggs (Figure 3). If we assume that larval emergence starts three to nine days after oviposition [9], it seems likely that foliar sprays were applied at the correct time and that timing was not the reason for poor efficacy.

The poor results of insecticides might be caused by the lack of insecticide efficacy against the cabbage maggot. Cabbage maggot control in Croatia is conducted using two organophosphorous insecticides, chlorpyrifos and dimethoate, and one neonicotinoid insecticide, imidacloprid [7]. Chlorpyrifos is widely used for the control of cabbage maggot larvae in brassica crops and could be considered as a standard treatment in numerous countries [19,30]. However, chlorpyrifos is not frequently used in Croatia because cabbage growers in Croatia usually do not have the necessary equipment to apply granular formulations of chlorpyrifos. After many years of use, it is suspected that cabbage maggot resistance to chlorpyrifos has developed in British Columbia (BC, Canada) [19], although this is yet to be confirmed by the Insecticide Resistance Action Committee (IRAC) [43]. The use of dimethoate either by spraying or by dipping of seedlings could be considered as the standard treatment against the cabbage maggot in Croatia. We did not find in the literature any supportive data on the strong efficacy of dimethoate, but growers in Croatia have used it for years with apparently acceptable control. Our results show some reduction in the average number of larvae/plant when dimethoate was applied either as a seedling dip or as a foliar spray but, the reduction was not significantly different from untreated plots (Figure 6). It is possible that the cabbage maggot adult, over years of dimethoate use in the area, has developed a level of resistance. If we take into account that 37 insect species already developed resistance to dimethoate [44] and that there are 60 reported cases of resistance development in the cabbage maggot to various other insecticides [44], it is a plausible scenario. However, until now, no resistance development to dimethoate has been reported in the cabbage maggot. Therefore, the possibility that the cabbage maggot has developed resistance to dimethoate in the region of Ogulin should be considered and investigated in the future.

Research conducted by different authors showed that imidacloprid did not reduce the number of cabbage maggot per plant [30,45] and therefore should not be used for the control of this pest in the United Kingdom [46]. However, imidaclopridbased products are officially allowed for cabbage maggot control in Croatia either by dipping of seedlings or by spraying. In some investigations, imidacloprid evidently resulted in an increase in the number of larvae per plant [46], as observed in our investigation after the foliar application of imidacloprid at a dose of 200 g a.i./ha (Figure 6). Finch and Edmonds [45] reported that the presence of imidacloprid in treated plants extended the period over which the cabbage maggot larvae continued to feed. Neonicotinoid insecticides, such as imidacloprid, act on the nervous system. In the laboratory, contact exposure of several economic species of wireworms to neonicotinoids prolonged periods of morbidity up to 150 days, characterized by the loss of coordination and inability to feed. Following this period of morbidity, wireworms generally recovered fully [47,48,49]. Although feeding by cabbage maggot larvae might be less intensive in the mentioned extended period due to loss of coordination, the final effect may be different. The effect of extended larval feeding on plant wilting might depend on climatic and agronomic conditions. If the conditions for plant regrowth are good, the final damage maybe will not be as visible as it would be in less favorable conditions. If larvae recover and finish their development, a population increase over a certain time period could be expected. A similar situation with wireworm populations in Canada was reported by Vernon *et al*. [50].

The best results were obtained by the use of thiamethoxam either through dipping of seedlings or as a foliar application. Acetamiprid had a certain effect on the percent of plants which showed symptoms of larval infestation, but it did not reduce the number of larvae per plant (Figure 6). Whether or not thiamethoxam or acetamiprid cause similar symptoms in larval behavior as imidacloprid is not clear. Although some researchers indicated that thiamethoxam may have a slightly different mode of action than imidacloprid [38], they agree that neonicotinoids act by binding to nicotinic acetylcholine receptors. In Croatia, thiamethoxam and acetamiprid are registered for foliar application for cabbage aphid control. Thiamethoxam is allowed for the dipping of seedlings for various vegetable species but it is not registered as a seedling dip for cabbage [7]. Šubić [6] reported that the efficacy of imidacloprid, thiamethoxam and acetamiprid-based insecticides applied by dipping seedlings is satisfactory for cabbage maggot control only if high doses of insecticides are applied. However, the application of high doses is not in accordance with IPM principles. Based on the fact that imidacloprid did not reduce the number of larvae per plant in all trials in this study, along with results obtained by other authors [45,46], we cannot support its use for cabbage maggot control. In the future, for cabbage maggot control in Croatia, the use of thiamethoxam should be considered, but further research is needed on possible application options. Siekmann and Hommes [51] reported good efficacy of thiamethoxam + abamectin as a seed treatment on cabbage maggot larvae when infestation occurred 6 weeks after sowing. They observed a slight loss of efficacy when plants were infested 11 weeks after sowing.

Seed treatment by different insecticides has been advocated as an effective control strategy against cabbage maggot larvae [21,30,45,46,51,52,53,54]. For seed treatment, various insecticides are recommended: chlorpyrifos [21,52,53], isofenphos [52], spinosad [30,51], thiamethoxam, clothianidin and abamectin [51], chlofenapyr, cyromazine and fipronil [52]. No significant negative effect ongermination was observed in either of these studies. Siekman and Hommes [51] and Jyoti *et al.* [52] reported that the activity of the seed treatment lasted 6 to 10 weeks after sowing, while Ester *et al.* [30] reported that the seed treatment could protect the plants from cabbage maggot larvae up to seven weeks after transplanting. In conditions that prevail in the region of Ogulin, cabbage maggot larvae start to infest cabbage plants at the earliest at the beginning of May, which is 5–7 weeks after transplanting (*i.e.*, 10 to 12 weeks after sowing). However, Šubić [6] reported an earlier infestation in the Croatian region of Međimurje. Since seed treatment with insecticides is not allowed for cabbage seeds in Croatia, the efficacy of seed treatment was not investigated in this study.

Although white cabbage in Croatia is considered a major crop, the total area is relatively small. Therefore, the limited number of active ingredients allowed for cabbage maggot control in Croatia is a result of the lack of interest of manufacturers in obtaining registration for cabbage. Due to high pesticide registration costs, manufacturers are not interested in registration. Hopefully joining the EU and accepting uniform principles of pesticide registration will change this situation.

A similar topic related to insecticide seed treatments was discussed by Suett [55], Finch and Collier [8], and Jukes *et al.* [46] who pointed out that the film-coating technique for treating seeds should be stimulated by appropriate legislative and political pressures. In the meantime, new findings related to the use of neonicotinoids as seed treatments and the fact that neonicotinoids have become an important environmental issue due their possible hazardous effect on bees and their unknown fate in the ecosystem [56,57,58,59] put the new question mark on the future use of neonicotinoids for seed treatment [60,61].

## 4. Conclusions

The main precondition for successful introduction of IPM is to develop systems in which all aspects of crop production and crop protection can be placed onto physiological rather than calendar time scales. This research provides practical insight into the population dynamics of the most important cabbage pest in the mountain region of Croatia, and, consequently, our results have important implications for improved management of this pest. Seasonal flight activity of the cabbage maggot during the cultivation season of the mid-early variety white cabbage in a mountain region of Croatia was correlated with DDA and Julian days and characterized by two peaks that correspond with two generations. The first peak began at 119 ± 7.5 Julian days and ended at 125.5 ± 8 Julian days. At that time, DDA reached 471.35 ± 74.97 °C. The second flight peak started at 172.8 ± 6.1 Julian days and ended at 179.3 ± 6.7 Julian days. At that time, DDA reached 1,217.28 ± 96.12 °C. The peak egg infestation was observed on 123 ± 6 Julian days. At that time DDAs were 412.31 ± 47.34 °C and the cumulative captures of flies were 124.11 ± 48.6 flies per yellow sticky trap. The peak larval infestation occurred on Julian day 146 ± 3. At that time, DDAs were 676.73 ± 57.8 °C and the cumulative captures of flies were 267.99 ± 79.64 flies per yellow sticky trap. The most accurate tool for predicting peak larval infestation is Julian days. If the cabbage maggot is controlled by foliar application of insecticides, the application should be conducted after peak egg occurrence and before peak larval infestation. Based on our results, in the area of Ogulin, this period is between 123 and 146 Julian days or when DDAs reach between 412.31 ± 47.34 and 676.73 ± 57.8 °C. Although our results agree generally with those of other workers, differences in degree-day and Julian day requirements occurred between the cabbage maggot in a mountain region of Croatia and those from geographically distant populations. Our study indicates that phenological determinations for the timing of peak periods of activity should be done on a regional basis. Population dynamics of this species should be therefore considered over a wide range of environmental, ecological and genetic influences. The DDA and Julian days are both appropriate forecasting methods by which we are able to predict the timing for insecticide application but, by these methods, we are not able to predict the need for pesticide application. Although by employing yellow sticky traps reliable data on the fly population can be obtained, the need for pesticide application requires additional information determined by direct methods based on “supervised control,” *i.e.*, a visual survey of the plants for eggs. Insecticides should be applied when the cumulative capture of flies reaches 100 flies/YST and when DDA are 400 °C. If only one trigger reaches the threshold, additional visual surveys should be employed to establish the infestation. Visual surveys of the plants for eggs is less precise than absolute sampling and therefore the proposed ET of 1–2 eggs/plant is acceptable for Croatian conditions, but further research on this topic is needed. The ET changes with the growth stage of the crop and, in Croatia, no data on this topic exist. The concept of having a threshold that marks the dividing line between two contrasting courses of action is the essential element for making decisions in IPM. Plant-growing conditions are very important and influence the crop damage at harvest. Good growing conditions enable plants to develop quickly and successfully even in the presence of larvae. Dimethoate and imidacloprid are registered for cabbage maggot control in Croatia but they are able to ensure only partial control, either applied as foliar treatment or through dipping the seedlings. Although some neonicotinoids showed good activity on cabbage maggot larvae (thiamethoxam) and some (imidacloprid) are recommended to be applied in combinations in order to broaden the spectrum of insects which can be controlled, future control tactics should be based on the use of more acceptable insecticides (*i.e.*, spinosad). The use of other insecticides should be discussed based on the new findings related to their possible impact on nontarget species and their environmental fate [56,57,58,59]. These strategies may include film-coating of brassica seeds. All other developments should be involved in cabbage maggot control as well. Insecticides may continue to play a role, but the timing of the application should be such as to prevent significant larval infestation after all other available methods have been employed.
